# Atomic-Scale Anisotropy Competition Drives Spin Reorientation in Intercalated 2D Ferromagnets

**DOI:** 10.1021/acsnanoscienceau.6c00043

**Published:** 2026-07-07

**Authors:** Yu-Sheng Tseng, Wan-Sheng Tang, Bao-Huei Huang, Yu-Hui Tang

**Affiliations:** Department of Physics, 34911 National Central University, Taoyuan City 320317, Taiwan

**Keywords:** magnetic anisotropy, spin−orbit torque, two-dimensional ferromagnetism, spin reorientation transition, spintronics, micromagnetic simulation

## Abstract

Precise control over magnetic anisotropy (MA) in two-dimensional (2D) van der Waals ferromagnets is essential for advancing next-generation spintronics; however, the atomic-scale mechanisms dictating intercalation-induced MA modulation remain largely unresolved. In this study, we elucidate the microscopic origins of the complex magnetic phase diagram in Cr-intercalated CrTe_2_ by integrating layer-resolved density functional theory-based spin–orbit torque (DFT-SOT) calculations with micromagnetic simulations. We uncover a concealed source of perpendicular magnetic anisotropy (PMA) localized within the intercalation layer, which directly competes with the robust surface-driven in-plane magnetic anisotropy (IMA). Macroscopically, this atomic-scale competition drives a thickness-dependent spin reorientation transition, wherein the volumetric accumulation of bulk-like PMA ultimately dominates over interfacial effects. Our findings propose a plausible microscopic framework for understanding the stoichiometry-dependent anisotropy in chromium chalcogenides, offering a promising theoretical perspective for exploring magnetic phases in 2D spintronic architectures.

## Graphical abstract

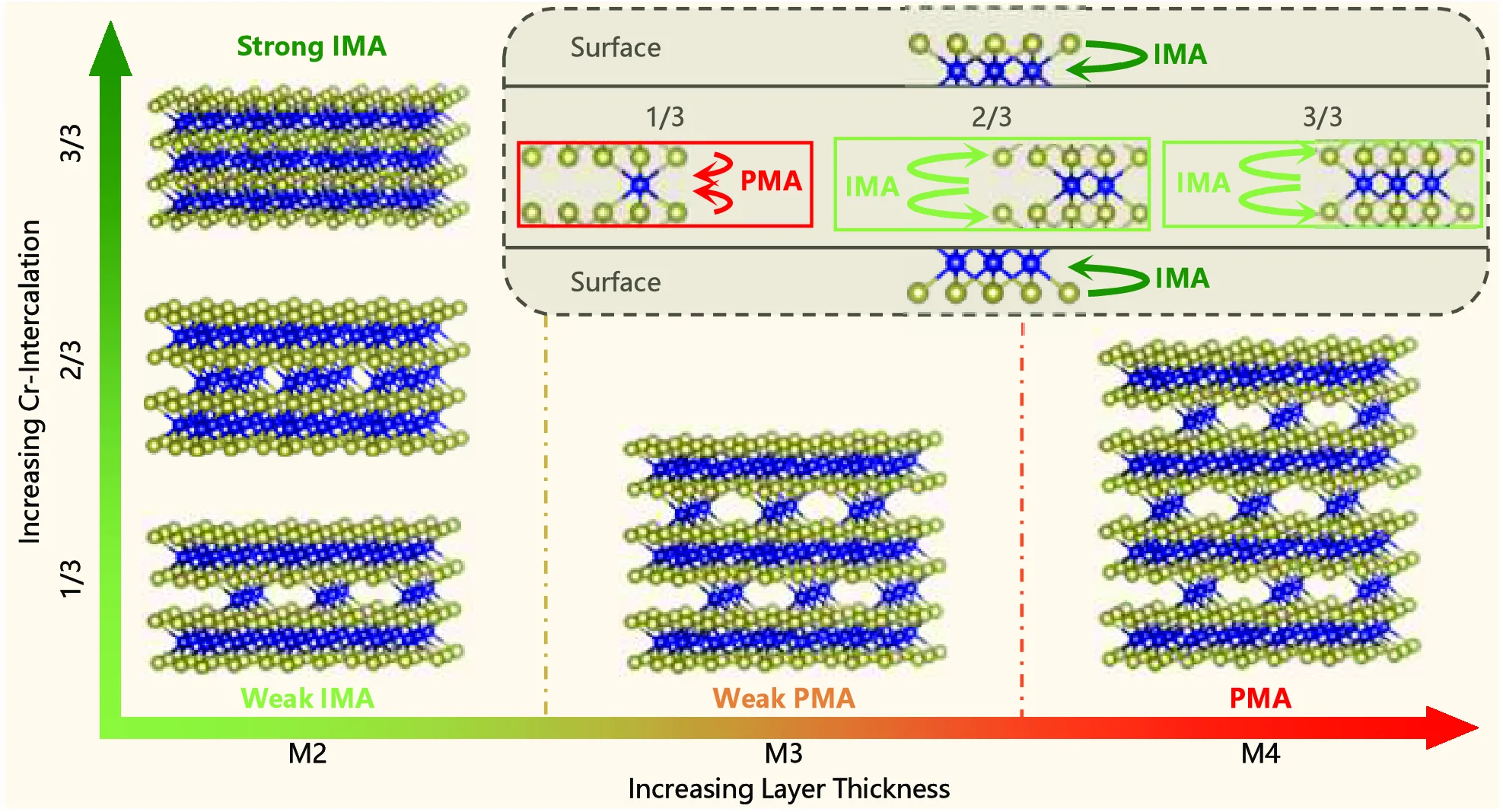

Two-dimensional (2D) van der Waals (vdW) ferromagnets have significantly advanced the field of spintronics, establishing a versatile platform where long-range magnetic ordering is sustained down to the atomic limit.^1–3^ A critical parameter determining the operational stability and energy efficiency of such nanoscale devices is magnetic anisotropy (MA), which dictates the preferential alignment of magnetic moments and serves as the foundation for nonvolatile memory technologies.^4–7^ Among the rapidly expanding family of 2D magnets, chromium chalcogenides have garnered intense focus due to their elevated Curie temperatures and unique itinerant ferromagnetic characteristics.^8–11^ Although chemical intercalation provides a highly effective degree of freedom to modulate these vdW materials beyond traditional strain engineering,^12–16^ the underlying microscopic mechanisms through which intercalation alters the magnetic ground state remain heavily debated.

Within the Cr–Te system specifically, self-intercalation acts as a vital control parameter for tuning both magnetic hardness and structural stability.^17–19^ Unlike the metastable CrTe_2_ phase,^20^ a variety of intercalated structural phases, including Cr_5_Te_8_, Cr_2_Te_3_, Cr_3_Te_4_, and CrTe, have been experimentally stabilized. Crucially, the magnetic anisotropy energy (MAE) in these systems demonstrates a highly complex, nonmonotonic dependence on both the Cr intercalation concentration and the film thickness. This complexity is highlighted by the coexistence of ferromagnetic and antiferromagnetic states in Cr_5_Te_8_,^21,22^ the recently observed canted ferromagnetic ground states in Cr_2_Te_3_ monolayers,^23^ and the dramatic anisotropy crossover documented in Cr_3_Te_4_.^24,25^ These observations suggest that the global MAE emerges from an intricate competition between intercalation-induced exchange coupling modifications and thickness-dependent surface anisotropies—a dynamic that transcends simple linear scaling.

A primary obstacle in decoding the behavior of these Cr–Te systems, particularly in the ultrathin regime, is the challenge of resolving competing magnetic interactions at the atomic scale. Conventional theoretical methodologies evaluating the spin reorientation transitions (SRT) between PMA and IMA typically rely on macrospin approximations.^26,27^ Such approximations, however, inherently obscure the nuanced, layer-specific contributions to the total MAE. In intercalated Cr–Te ultrathin films, the broken spatial symmetry at the surface presents a fundamentally different local environment compared to the interior intercalation layers. Consequently, traditional analytical approaches fail to capture how concealed local torques—driven by the interplay of spin–orbit coupling (SOC) and interlayer spin transfer—either compete or synergize to define the global magnetic ground state.

In this Letter, we bridge the critical gap between atomic-scale magnetic interactions and macroscopic magnetic phenomena in CrTe_2_–Cr_δ_–CrTe_2_ ultrathin films (δ = 1/3, 2/3, and 1). By implementing a multiscale modeling paradigm that integrates layer-resolved DFT-SOT calculations^28,29^ with micromagnetic simulations, we decouple the microscopic antagonisms frequently masked by macrospin models. We report the identification of a hidden source of PMA stemming exclusively from the intercalation layer, which vigorously competes with the predominant surface-driven IMA. Unveiling this layer-specific mechanism offers a compelling atomic-scale scenario for the thickness-dependent SRTs, laying down a qualitative foundation for exploring magnetic ground states via precise stoichiometric modulation.

The magnetization dynamics is described by the macroscopic torque equation $T^{M}\equiv \frac{1}{\gamma }\frac{dM}{dt}=-M\times B^{e\mathrm{ff}}$ , where γ denotes the gyromagnetic ratio and **B** ^eff^ represents the effective magnetic field. The magnetic torque, defined as **T** ^M^(θ) ≡ *∂*MAE­(θ)/*∂θ*, corresponds to the angular derivative of the magnetic anisotropy energy, MAE = *E*_tot_(θ) – *E*_tot_(0). Alternatively, to execute the DFT-SOT calculation with noncollinear magnetic configurations, the magnetic direction of all atomic moments was rotated around the *x* axis with respect to the *z* axis by an angle θ. For each angle θ, the self-consistent device Hamiltonian was used to evaluate the exchange spin torque (**T** ^XC^), the local spin–orbit torque (**T** ^SO^), and the spin accumulation (**Φ**) injected from the neighboring orbitals. The *net* spin torque acting on the magnetic moment is defined by **T** ^M^ = **T** ^XC^ = – **T** ^SO^ – **Φ**.

Figure 1a,b presents the angle-dependent MAE and **T** ^M^, respectively, for the Cr-intercalated CrTe_2_–Cr_δ_–CrTe_2_ ultrathin films (δ = 1/3, 2/3, and 1). The clear sinusoidal profile of the MAE confirms that all investigated compositions possess IMA, with the global energy minimum pinned at θ = 90°. Furthermore, the minimum MAE values demonstrate excellent quantitative parity with the **T** ^M^ magnitudes calculated at θ = 45°. As schematically shown in Figure 1c, any deviation of the magnetization **M** from the easy axis induces an effective field oriented along the −*z* direction. This induced field produces a restoring torque **T** ^M^ that continuously drives **M** back toward the in-plane easy axis, yielding **T** ^M^ < 0 for 0° < θ < 90° and **T** ^M^ > 0 for 90° < θ < 180°.

**Figure 1:**
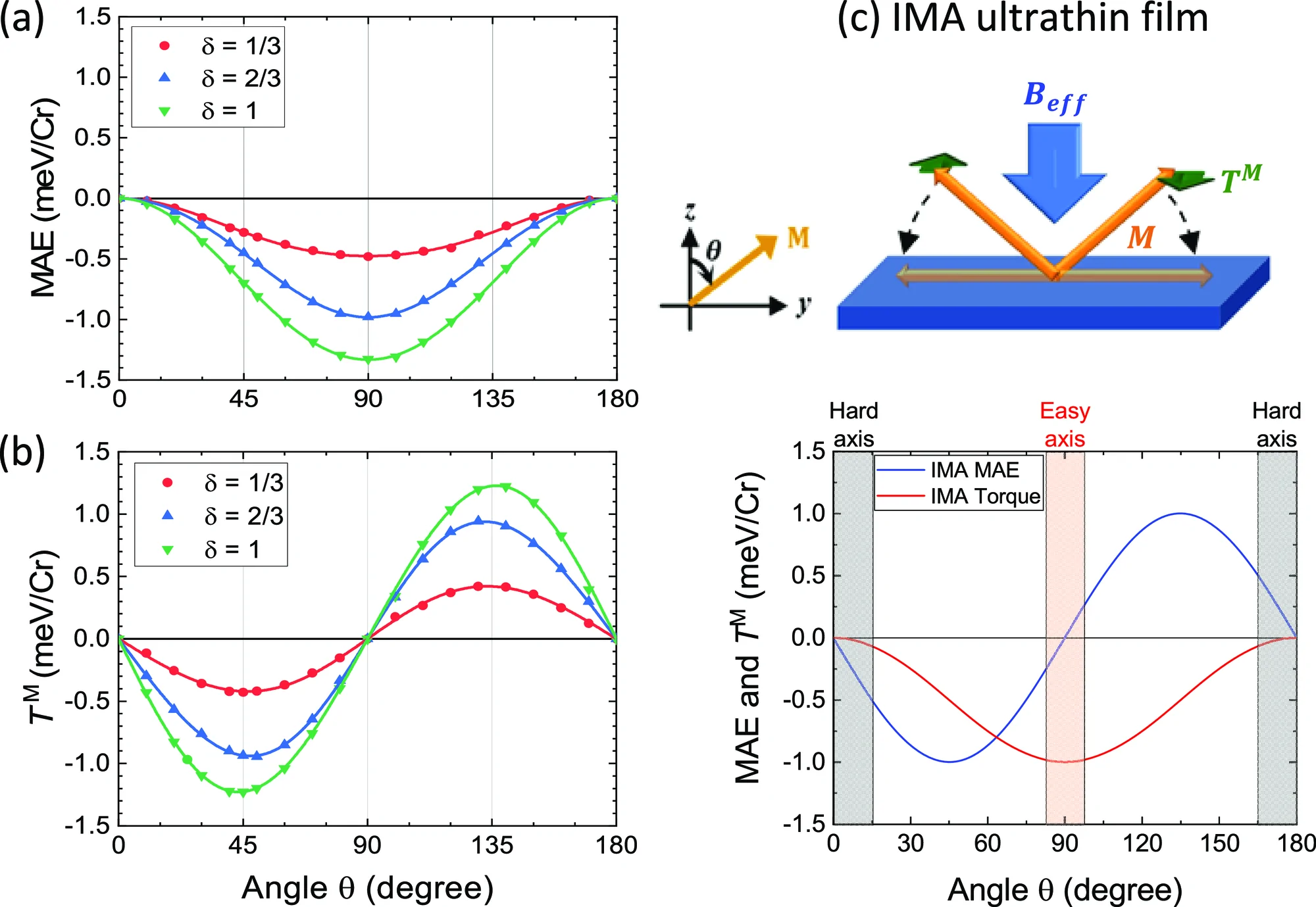
(a) Angle-dependent magnetic anisotropy energy (MAE) and (b) DFT-SOT calculated spin torque **T** ^M^. (c) Schematic of the coordinate system: magnetization **M** is rotated by an angle θ from the *z* axis. All cases exhibit in-plane magnetic anisotropy (IMA).

Crucially, the overall MAE is significantly attenuated upon Cr intercalation, as shown in Figure 1a, with the lowest Cr concentration (δ = 1/3) displaying the weakest IMA. To elucidate the physical link between Cr intercalation and this suppressed IMA, we apply the Heisenberg exchange model. This model characterizes the total energy difference when two magnetic moments (**M**_*i*_ and **M**_*j*_) are aligned parallel (P) versus antiparallel (AP), governed by *E*_tot_ = *E*_0_ – ∑_*ij*_ *J*_*ij*_(**M**_*i*_·**M**_*j*_), where *E*_0_ is the nonmagnetic baseline energy and *J*_*ij*_ > 0 (*J*_*ij*_ < 0) is the exchange coupling parameter for the P (AP) state. For the three intercalation scenarios (δ = 1/3, 2/3, and 1), we partition the exchange coupling parameters into intralayer (*J*_A_, *J*_B_, *J*_C_) and interlayer (*J*_D_, *J*_E_, *J*_F_) contributions. The extraction methodology for these fitted parameters is fully outlined in Supporting Information (SI) section S-III and Figure S2. The derived *J* parameters are plotted in Figure 2a,b, and their respective spatial mappings are illustrated in Figure 2c,d. Notably, only the δ = 1/3 configuration yields a negative *J*_D_, indicating an energetic preference for antiparallel (AP) alignment between that specific pair of Cr atoms (highlighted by the red square). Conversely, all other extracted *J* values remain strictly positive across the three concentrations, denoting standard ferromagnetic (P) ordering. Therefore, at this low intercalation density (δ = 1/3), the unique coexistence of a singular antiparallel interlayer coupling (*J*_D_ < 0) alongside parallel intra/interlayer couplings (*J*_A,B,E_ > 0) generates a highly competitive magnetic interaction environment. This intrinsic magnetic frustration directly accounts for the minimized IMA observed in Figure 1a, as the macroscopic MAE merely reflects the net summation of these deeply opposed local anisotropy contributions.

**Figure 2:**
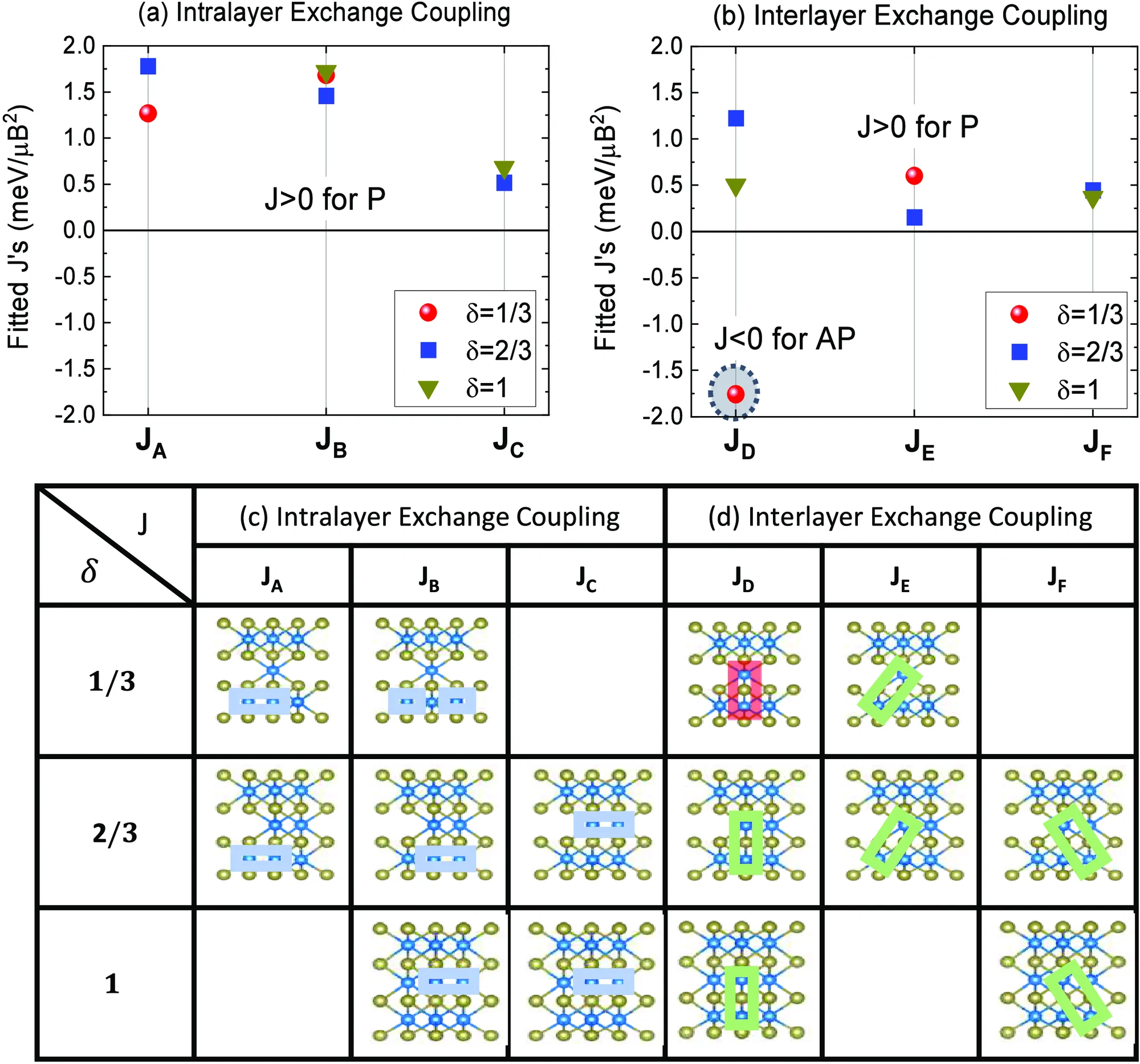
(a) Fitted intralayer exchange couplings (*J*_A_, *J*_B_, *J*_C_) and (b) fitted interlayer exchange couplings (*J*_D_, *J*_E_, *J*_F_) for CrTe_2_–Cr_δ_–CrTe_2_ ultrathin films (δ = 1/3, 2/3, and 1). (c, d) Spatial distributions used to categorize (c) intralayer exchange couplings and (d) interlayer exchange coupling for the three Cr-intercalation cases. The positive and negative *J* values imply that a pair of Cr atoms (as indicated by the color square) prefer the parallel (P) and antiparallel (AP) alignments, respectively.

To pinpoint the exact microscopic function of the intercalated Cr, we executed layer-resolved DFT-SOT analyses. Figure 3a–c profiles the layer-decomposed components **T**_*i*_^M^, −**T**_*i*_^SO^, and −**Φ**_*i*_ at θ = 45° for δ = 1/3, 2/3, and 1, respectively. The *net* magnetic torque (**T**_*i*_^M^) exerted on the local moment (**M**_*i*_) of layer *i* is formulated as the superposition of the local spin–orbit torque (−**T**_*i*_^SO^) and the spin-transfer torque resulting from interlayer spin exchange (−**Φ**_*i*_). Strikingly, only the low-concentration regime (δ = 1/3) reveals a vivid coexistence of opposing anisotropies: the central intercalated Cr-I layer strongly favors PMA (**T**_Cr‑I_^M^ > 0), while the enclosing top and bottom Cr-M monolayers mandate IMA (**T**_Cr‑M_^M^ < 0). At higher intercalation densities (δ = 2/3 and 1), this competition vanishes, and all magnetic Cr layers unanimously support IMA.

**Figure 3:**
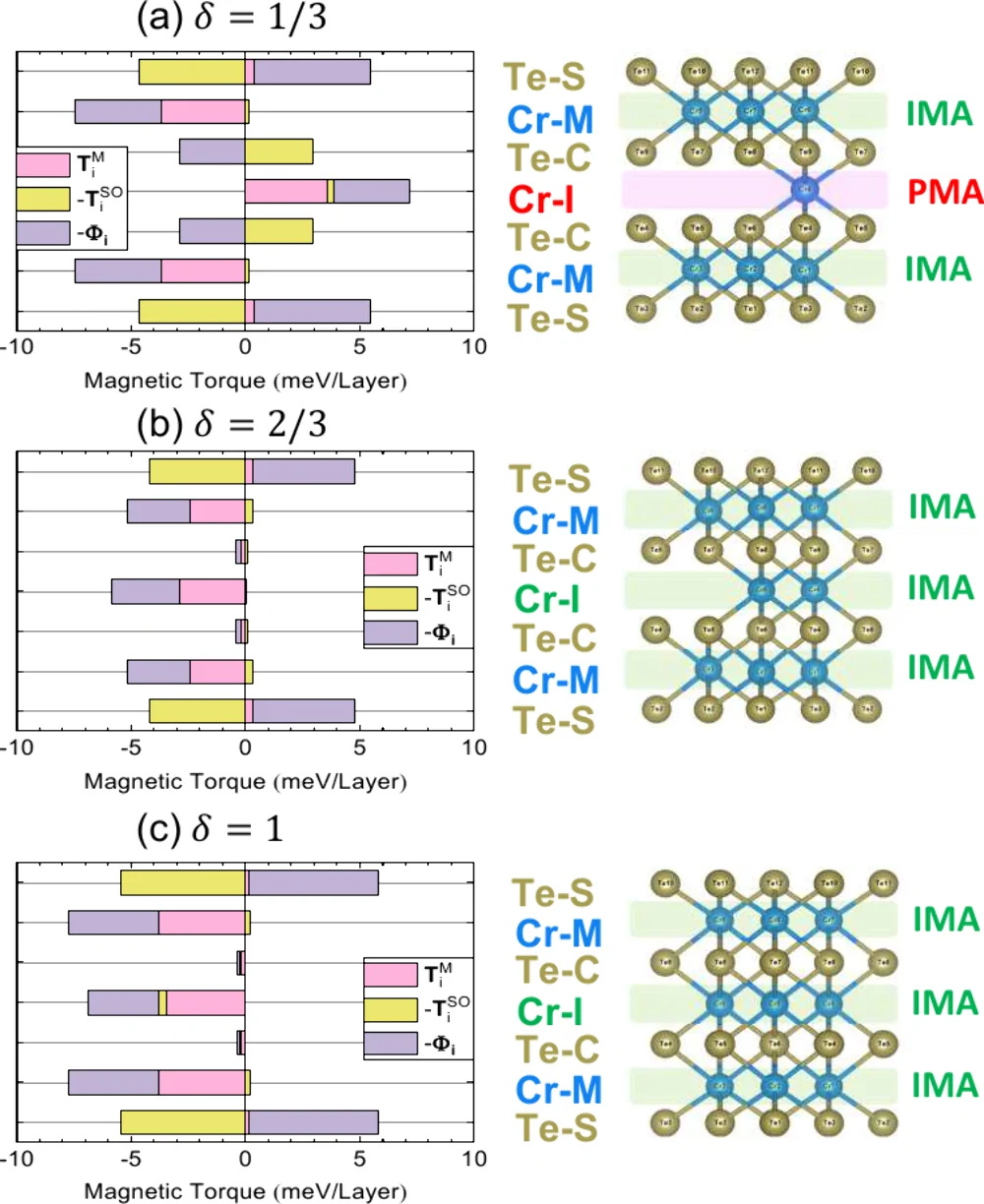
Layer-resolved SOT components at θ = 45° for (a) δ = 1/3, (b) δ = 2/3, and (c) δ = 1. The net magnetic torque on layer *i* is decomposed as **T**_*i*_^M^ = −**T**_*i*_^SO^ – **Φ**_*i*_. Pink and green shaded regions denote contributions favoring PMA and IMA, respectively.

Consequently, the mixed magnetic anisotropy characterizing the δ = 1/3 system is not the product of a unified interaction, but rather the consequence of aggressively competing local anisotropies. Analyzing the layer-resolved −**T**_*i*_^SO^ and −**Φ**_*i*_ terms uncovers an atomic-scale tug-of-war, graphically summarized in Figure 4. On one front, the pronounced interfacial spin–orbit coupling (iSOC) at the exterior Te layers (Te-S) (Figure 3a) generates a powerful local torque (−**T**_Te‑S_^SO^ ≈ −5 meV). This surface-localized torque induces a spin current that strips the out-of-plane magnetic moment (+*m*_*z*_) from the adjacent Cr-M layers (−**Φ**_Cr‑M_ ≈ −4 meV), strongly enforcing IMA (green arrows in Figure 4a). Concurrently, the interior Te layers (Te-C)—characterized by partially unsaturated dangling bonds—generate an opposing torque (−**T**_Te‑C_^SO^ ≈ +3 meV). This interior torque propels a reverse spin transfer (−**Φ**_Cr‑I_ ≈ +3 meV) directly into the intercalation layer (pink curved arrows in Figure 4a), actively cultivating a hidden reservoir of PMA (**T**_Cr‑I_^M^ > 0). The overarching magnetic ground state for δ = 1/3 is therefore determined by this intense standoff between surface-propagated IMA and intercalation-nurtured PMA.

**Figure 4:**
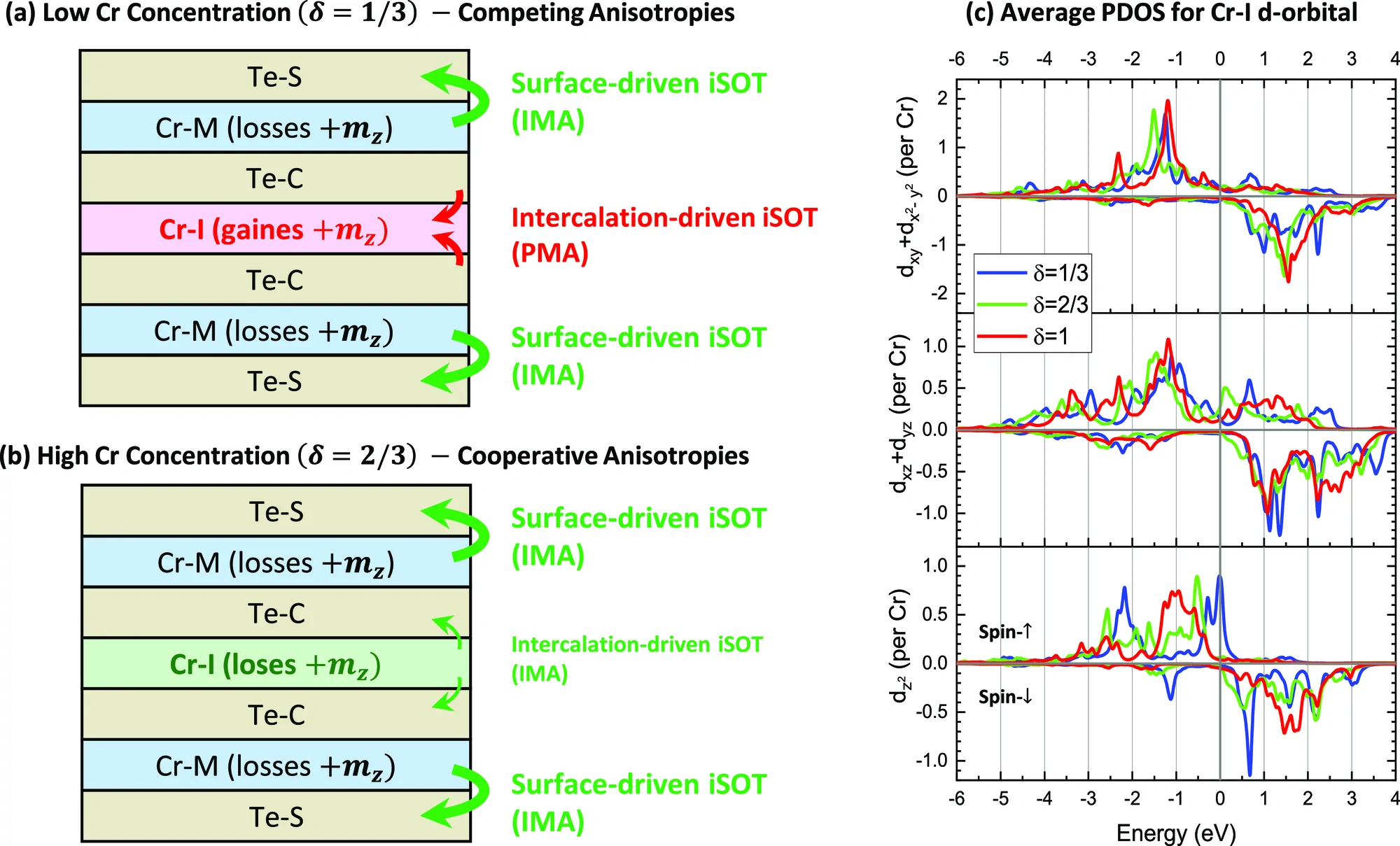
Microscopic origin of the anisotropy mechanisms. (a) For δ = 1/3 (competing anisotropies), surface-driven IMA (green arrows) competes with hidden intercalation-driven PMA (red arrows). (b) For δ = 2/3 (cooperative anisotropies), both surface and intercalation contributions favor IMA. Curved arrows indicate the direction of interlayer spin currents. (c) Orbital-resolved projected density of states (PDOS) for the intercalated Cr-I d orbitals (d_*xy*_ + d_*x* ^2^+*y* ^2^_, d_*xz*_ + d_*yz*_, and d_*z* ^2^_) under varying Cr concentrations δ.

In sharp contrast, elevating the Cr concentration to δ = 2/3 completely restructures this interplay, shifting the system from a competitive to a cooperative anisotropy regime. To elucidate the microscopic origin of this transition and the fundamental role of electronic structure, we plot the orbital-resolved projected density of states (PDOS) for the intercalated Cr-I atoms in Figure 4(c). In the low-concentration regime (δ = 1/3), a pronounced, highly localized peak in the out-of-plane d_*z* ^2^_ orbital resides precisely at the Fermi level (*E*_F_ = 0 eV). This localized state arises from incomplete hybridization with the adjacent interior Te atoms, providing the primary channel that generates the hidden PMA. However, as the Cr concentration increases (δ ≥ 2/3), the enhanced Cr occupancy fully passivates the Te–C dangling bonds through the p_*z*_–d_*z* ^2^_ orbital hybridization. Consequently, the d_*z* ^2^_ states broaden and shift significantly to lower energies (higher binding energies); therefore, the removal of these localized d_*z* ^2^_ states from *E*_F_ effectively neutralizes the microscopic pathways responsible for the hidden PMA. With this pathway quenched, the intercalation-driven torque (**T**_Cr‑I_^M^ ≈ −3 meV) is forced into alignment with the dominant surface IMA. Ultimately, this cooperative synchronization between surface and intercalation torques (green curved arrows in Figure 4b) securely anchors the global in-plane magnetic state. Comprehensive procedures for the spin current flow analysis are available in SI section S-IV and Figure S3.

We subsequently isolate the low Cr concentration state (δ = 1/3, designated as the M2 Cr_2_Te_3_ ultrathin film) to demonstrate how this atomic competition shapes the macroscopic magnetic hysteresis loop across the SRT. Micromagnetic simulations were executed utilizing MuMax3,^30^ implementing a distinct three-layer architecture: a central intercalation layer (Cr-I) hosting PMA, bounded by two surface layers (top and bottom Cr-M) exhibiting IMA. Both the saturation magnetization (*M*_sat_) and the effective uniaxial anisotropy constant (*K*_u_) were extracted directly from the layer-resolved DFT-SOT data at θ = 45°. Crucially, rather than employing empirical assignments, the macroscopic exchange stiffness constants were strictly mapped from our DFT-calculated microscopic Heisenberg exchange parameters. This exact translation yields an intralayer exchange stiffness (*A*_intra_ = 1.35 pJ/m) and a geometrically frustrated, much softer interlayer exchange stiffness (*A*_inter_ = 0.13 pJ/m). All fundamental simulation parameters and the multiscale mapping derivations are comprehensively detailed in SI section S-V and Tables S2–S5.

To explicitly visualize the influence of the hidden intercalation-driven PMA in the δ = 1/3 system, we simulated the magnetic hysteresis loop under an applied out-of-plane field (*B*_*z*_), capturing a clearly defined four-stage SRT progression, as depicted in Figure 5b. In the robust high-field regime (*B*_*z*_ = 8.0 to 5.7 T), the external field forcibly aligns both the PMA and IMA layers along the +*z* axis (stage 1). As the external field weakens (5.7 to −3.0 T), it remains sufficient to anchor the PMA layer but can no longer pin the IMA layers, causing them to rotate smoothly from +*z* toward −*z* (stage 2). Importantly, the spatial proximity of the PMA layer prevents the IMA layers from undergoing a complete, abrupt rotation; consequently, the intrinsic coercivity of the IMA layers is suppressed due to the mediating weak exchange stiffness. Near −3.0 T, the central PMA layer undergoes a definitive flip from +*z* to −*z* (stage 3). In a reciprocal manner, the coercivity of the PMA layer is dynamically reduced by the surrounding IMA layers through this same delicate interlayer coupling. Promptly following the PMA flip, the IMA layers finalize their rotation to align fully with the −*z* direction (stage 4). Ultimately, all three Cr layers exert a profound mutual influence mediated by the finite interlayer exchange coupling, which systematically diminishes the coercive fields of each individual layer. Correspondingly, under an applied in-plane field (*B*_*x*_) (shown in Figure 5c), while the top and bottom IMA layers dictate the primary switching behavior, the embedded PMA layer fundamentally lowers the system’s overall coercivity via these interlayer exchange interactions.

**Figure 5:**
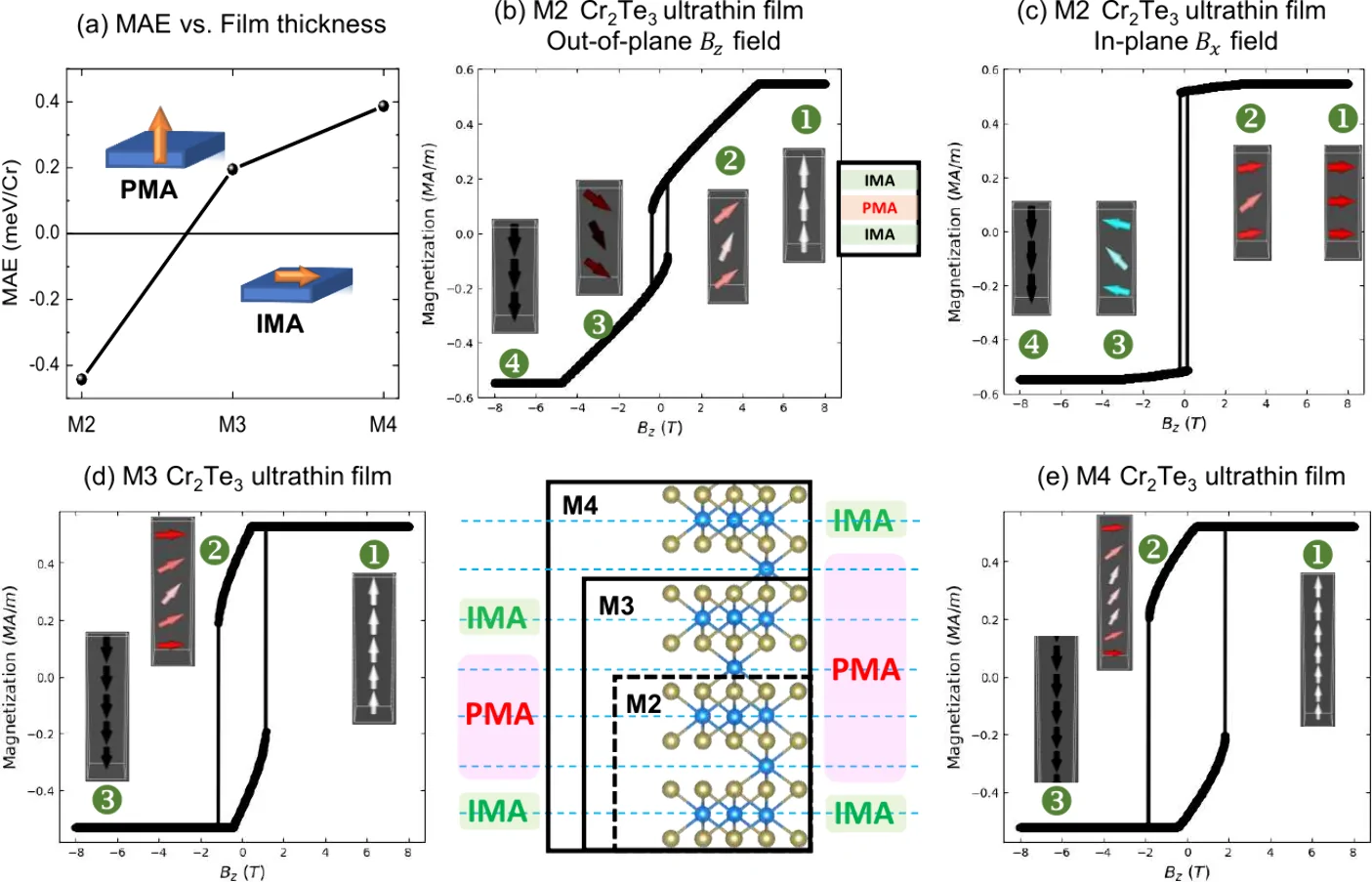
Thickness-dependent spin reorientation in Cr_2_Te_3_ (δ = 1/3) ultrathin films. (a) Total magnetic torque **T** ^M^ as a function of thickness, revealing the crossover from IMA to PMA driven by the accumulation of bulk-like interior layers. (b, c) Simulated magnetic hysteresis loops for the δ = 1/3 case (denoted as the M2 Cr_2_Te_3_ ultrathin film) under (b) out-of-plane (*B*_*z*_) and (c) in-plane (*B*_*x*_) external fields. (d, e) Simulated magnetic hysteresis loops for (d) M2 and (e) M3 Cr_2_Te_3_ ultrathin films under out-of-plane (*B*_*z*_) external fields. The simulation employs a three-layer model where a central PMA intercalation layer is sandwiched between two IMA surface layers. The layer-resolved SOT components at θ = 45° for M3 and M4 cases are presented in Figure S4.

Finally, we evaluate the thickness-dependent MA in Cr_2_Te_3_ (δ = 1/3) ultrathin films by expanding our model to the M3 (five Cr layers) and M4 (seven Cr layers) configurations, detailed in Figure 5. A decisive crossover from global IMA to global PMA is evident in the calculated total magnetic torque (Figure 5a). Our layer-resolved DFT-SOT analysis (bottom panels of Figure 5a,c) confirms that this SRT is driven by the robust persistence of intercalation-driven PMA within the newly added interior Cr layers. This structural evolution instigates a direct volumetric competition between the surface-bound IMA and the bulk-like interior PMA. As the film thickness scales, the cumulative volumetric weight of the interior PMA eventually overrides the constraints of the surface IMA. Simulated magnetic hysteresis loops under *B*_*z*_ substantiate this PMA-dominated switching behavior in thicker films (Figure 5d,e). This macroscopic scaling dynamics offers a plausible qualitative explanation for the thickness-tunable SRTs documented in recent empirical studies.^23^ While real experimental systems may encompass additional complex factors such as defects or substrate effects, our model qualitatively links the transition to the volumetric expansion of the bulk-like intercalation phase against fixed surface boundary conditions.

In conclusion, we have decoded the microscopic origins governing magnetic anisotropy modulation in Cr-intercalated CrTe_2_ ultrathin films. Utilizing a multiscale layer-resolved DFT-SOT framework, we illustrate that the magnetic ground state is dictated by an intense, antagonistic interplay between highly localized, layer-specific torques. We reveal a concealed source of PMA residing within the intercalation layer that directly competes with the dominant surface-driven IMA, establishing a volume-dependent scaling law that successfully drives a spin reorientation transition with increasing film thickness. This framework provides a promising physical mechanism for the intricate stoichiometry-dependent anisotropies frequently observed in chromium chalcogenides, offering a coherent theoretical perspective to interpret the complex behaviors in 2D magnetism.

From a broader perspective, the discovery of this hidden PMA source carries significant implications for stabilizing exotic topological spin textures. The emergent Dzyaloshinskii–Moriya interaction (DMI)—triggered by intercalation-induced structural symmetry breaking—demands a robust, competing PMA to host and sustain Néel-type skyrmions. Our theoretical model implies that the highly localized PMA generated within these intercalation layers may deliver the precise energetic equilibrium required to stabilize such skyrmionic phases at elevated temperatures. This potent synergy between intercalation-driven DMI and hidden PMA highlights the exceptional promise of Cr_2_Te_3_ for deployment in high-density racetrack memories and next-generation neuromorphic computing architectures.^16,31^ We envision this comprehensive multidisciplinary framework acting as a foundational predictive tool for mapping complex spin dynamics and engineering topological transitions across a broad spectrum of van der Waals heterostructures.

## Supplementary Material



## Data Availability

The data that support the findings of this study are available from the corresponding author upon reasonable request.

## Notes

Structural relaxation, DFT-SOT method, Heisenberg exchange model, spin-transfer analysis, and micromagnetic simulation methodology (PDF)
