# Supplementary material for: Atomic-Scale Anisotropy Competition Drives Spin Reorientation in Intercalated 2D Ferromagnets
Source: ACS Nanosci Au. 2026 Jul 7;6(5):850–6. doi: 10.1021/acsnanoscienceau.6c00043 (PMC13692741; doi:10.1021/acsnanoscienceau.6c00043)
Supplement: Supplementary file 1 [file ng6c00043_si_001.pdf]

Atomic-Scale Anisotropy Competition Drives Spin Reorientation in Intercalated 2D Ferromagnets

Yu-Sheng Tseng,[1,*] Wan-Sheng Tang,[1,*] Bao-Huei Huang,[1] and Yu-Hui Tang[1,†]

[1]*Department of Physics, National Central University, Taoyuan City 320317, Taiwan*

S-I. STRUCTURAL RELAXATION

The structural relaxation of all $CrTe_2-Cr_\delta-CrTe_2$ ultrathin films, specifically for δ = 0, 1/3, 2/3, and 1, was performed using the Vienna Ab Initio Simulation Package (VASP) [1–3] with the Perdew-Burke-Ernzerhof (PBE) generalized gradient approximation (GGA) exchange-correlation functional. The top and side views of the four optimized structures are presented in Figure S1. The optimized in-plane lattice constant (a), the three interlayer distances (Δz_1, Δz_2, Δz_3), and the average magnetic moments are listed in Table S1.

To simulate an isolated ultrathin film, a vacuum layer of approximately 15 Å was introduced in the z-direction between adjacent films. The calculations utilized a Γ-centered k-point grid of $12 \times 12 \times 1$ and a plane-wave kinetic energy cutoff of 500 eV. Convergence was achieved when the energy difference between self-consistent field (SCF) cycles was less than 10^{-6} eV, and the residual force on each atom was constrained to be below 0.01 eV/Å. Furthermore, to accurately optimize the interlayer distances, van der Waals (vdW) interactions were included using the optB86b-vdW exchange functional.

δ	a	Δ_{z1}	Δ_{z2}	Δ_{z3}	M_{Cr-M}	M_{Cr-I}	M_{Te-S}	M_{Te-C}
0	6.501	3.17	2.91	3.17	2.9	2.9	-0.2	-0.2
1/3	6.688	3.07	2.91	3.07	3.1	3.0	-0.2	-0.2
2/3	6.766	3.08	3.10	3.08	3.3	3.4	-0.3	-0.1
1	6.837	3.06	3.16	3.06	3.4	3.6	-0.3	-0.1

TABLE S1. Optimized lattice constants, a (Å), three interlayer distances, $\Delta z_{1,2,3}$ (Å), and average magnetic moments (μ_B) for $CrTe_2$-Cr_δ-$CrTe_2$ ultrathin films. The labels are denoted in Figure S1.

* These authors contributed equally to this work.

† yhtang@cc.ncu.edu.tw

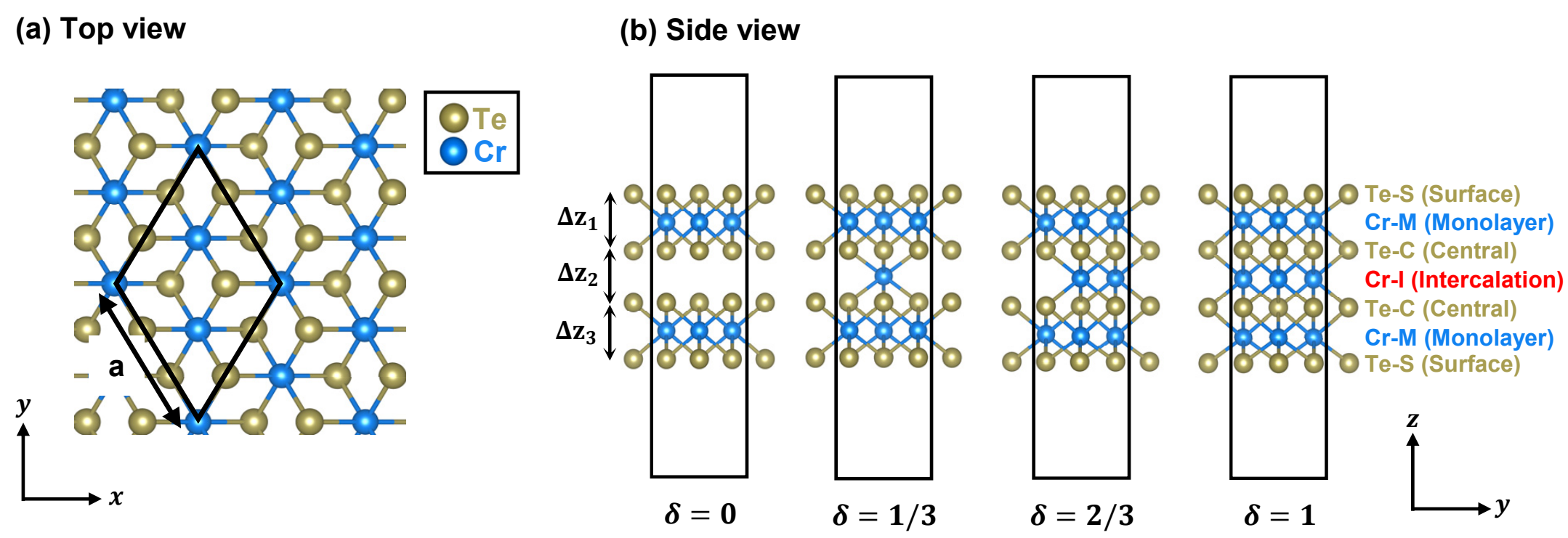

FIG. S1. (a) Top view and (b) side views of $CrTe_2-Cr_\delta-CrTe_2$ ultrathin films with δ = 0, 1/3, 2/3, and 1. The optimized in-plane lattice constants, a, and three interlayer distances ($\Delta z_{1,2,3}$) are listed in Table S1.

S-II. DFT-BASED SPIN ORBIT TORQUE (DFT-SOT) METHOD

The self-consistent electronic structure was obtained using the Nanodcal package [4, 5], which combines Density Functional Theory (DFT) with the Non-Equilibrium Green's Function (NEGF) formalism and incorporates spin-orbit coupling (SOC). The Hamiltonian and overlap matrices were constructed using a double-ζ double-polarized linear combination of atomic orbitals (LCAO) basis set, and the Local Spin Density Approximation (LSDA) with the PZ81 functional.

Given the self-consistent Hamiltonian, our self-developed JunPy package [6, 7], which integrates first-principles calculations with the NEGF formalism, facilitates general and universal calculations of both spin torque and spin current accumulation. Since the calculated magnetic torques are on the order of meV/atom, a stringent convergence criterion of 10^{-6} eV was set for both the Hamiltonian and the total energy. The energy cutoff and the cutoff length for k-space were also enlarged to 300 Hartree and 160 Bohr, respectively. The Monkhorst-Pack k-point samplings used in the Nanodcal and JunPy calculations were $27 \times 27 \times 1$ and $65 \times 65 \times 1$, respectively.

Although incorporating a Hubbard U correction is often crucial for capturing the correlated electronic structure and the localized d-bands in transition-metal chalcogenides (e.g., U=1.7~3.0 eV [8, 9]), it was not included in our present NEGF calculations. To achieve self-consistent energy convergence under the highly stringent criteria required for evaluating this system (μeV), while simultaneously incorporating full SOC and non-collinear magnetism, introduces numerical challenges that exceed current standard computational capabilities. Thus, to ensure robust self-consistent convergence and to obtain a conceptually reliable angle dependence of the layer-resolved

magnetic anisotropy contribution, the DFT-SOT calculations were performed at the standard DFT level without the Hubbard U correction.

To execute the DFT-SOT calculation with noncollinear magnetic configurations, the magnetic direction of all atomic moments was rotated around the x axis with respect to the z axis by an angle θ. For each angle θ, the self-consistent device Hamiltonian was used to evaluate the exchange spin torque ($\mathbf{T}_{\lambda}^{\mathrm{XC}}$), the local spin-orbit torque ($\mathbf{T}_{\lambda}^{\mathrm{SO}}$), and the spin accumulation ($\mathbf{\Phi}_{\lambda}$) injected from the neighboring orbitals λ'. The *net* spin torque acting on the magnetic moment of orbital λ is defined by: $\mathbf{T}_{\lambda}^{\mathrm{M}} = \mathbf{T}_{\lambda}^{\mathrm{XC}} = -\mathbf{T}_{\lambda}^{\mathrm{SO}} - \mathbf{\Phi}_{\lambda}$.

S-III. HEISENBERG EXCHANGE MODEL

The Heisenberg exchange model describes the total energy difference between two magnetic moments ($\mathbf{M}_i$ and $\mathbf{M}_j$) aligned parallel (P) or antiparallel (AP), given by $E_{tot} = E_0 - \Sigma_{ij} J_{ij} (\mathbf{M}_i \cdot \mathbf{M}_j)$, where E_0 is the system energy without exchange interaction and $J_{ij}>0$ ($J_{ij}<0$) is the exchange coupling parameter for P (AP) configuration. For the three Cr-intercalation cases ($\delta = 1/3$, 2/3, and 1), the exchange coupling parameters can be grouped into intralayer couplings (J_A, J_B, J_C) and interlayer couplings (J_D, J_E, J_F) as depicted in Figures 2(c) and 2(d), respectively.

- For $\delta = 1/3$ (J_A, J_B, J_D, J_E),

$$\begin{aligned} E = E_0 &- J_A\big(3\,\mathbf{M}_1 \cdot \mathbf{M}_2 + 3\,\mathbf{M}_1 \cdot \mathbf{M}_3 + 3\,\mathbf{M}_5 \cdot \mathbf{M}_6 + 3\,\mathbf{M}_6 \cdot \mathbf{M}_7\big) \\ &- J_B\big(\mathbf{M}_1 \cdot \mathbf{M}_4 + \mathbf{M}_4 \cdot \mathbf{M}_6\big) \\ &- J_D\big(3\,\mathbf{M}_2 \cdot \mathbf{M}_3 + 3\,\mathbf{M}_5 \cdot \mathbf{M}_7\big) \\ &- J_E\big(3\,\mathbf{M}_2 \cdot \mathbf{M}_4 + 3\,\mathbf{M}_3 \cdot \mathbf{M}_4 + 3\,\mathbf{M}_4 \cdot \mathbf{M}_5 + 3\,\mathbf{M}_4 \cdot \mathbf{M}_7\big) \end{aligned}$$

- For $\delta = 2/3$ (J_A, J_B, J_C, J_D, J_E, J_F),

$$\begin{aligned} E = E_0 &- J_A\big(3\,\mathbf{M}_1 \cdot \mathbf{M}_3 + 3\,\mathbf{M}_2 \cdot \mathbf{M}_3 + 3\,\mathbf{M}_6 \cdot \mathbf{M}_7 + 3\,\mathbf{M}_6 \cdot \mathbf{M}_8\big) \\ &- J_B\big(3\,\mathbf{M}_1 \cdot \mathbf{M}_2 + 3\,\mathbf{M}_7 \cdot \mathbf{M}_8\big) \\ &- J_C\big(3\,\mathbf{M}_4 \cdot \mathbf{M}_5\big) \\ &- J_D\big(\mathbf{M}_1 \cdot \mathbf{M}_4 + \mathbf{M}_2 \cdot \mathbf{M}_5 + \mathbf{M}_4 \cdot \mathbf{M}_7 + \mathbf{M}_5 \cdot \mathbf{M}_8\big) \\ &- J_E\big(3\,\mathbf{M}_3 \cdot \mathbf{M}_4 + 3\,\mathbf{M}_3 \cdot \mathbf{M}_5 + 3\,\mathbf{M}_4 \cdot \mathbf{M}_6 + 3\,\mathbf{M}_5 \cdot \mathbf{M}_6\big) \\ &- J_F\big(3\,\mathbf{M}_1 \cdot \mathbf{M}_5 + 3\,\mathbf{M}_2 \cdot \mathbf{M}_4 + 3\,\mathbf{M}_4 \cdot \mathbf{M}_8 + 3\,\mathbf{M}_5 \cdot \mathbf{M}_7\big) \end{aligned}$$

- For $\delta = 1$ (J_A, J_B, J_C, J_D, J_E, J_F),

$$\begin{aligned} E = E_0 &- J_B\big(3\,\mathbf{M}_1 \cdot \mathbf{M}_2 + 3\,\mathbf{M}_1 \cdot \mathbf{M}_3 + 3\,\mathbf{M}_2 \cdot \mathbf{M}_3 + 3\,\mathbf{M}_7 \cdot \mathbf{M}_8 + 3\,\mathbf{M}_7 \cdot \mathbf{M}_9 + 3\,\mathbf{M}_8 \cdot \mathbf{M}_9\big) \\ &- J_C\big(3\,\mathbf{M}_4 \cdot \mathbf{M}_5 + 3\,\mathbf{M}_4 \cdot \mathbf{M}_6 + 3\,\mathbf{M}_5 \cdot \mathbf{M}_6\big) \\ &- J_D\big(\mathbf{M}_1 \cdot \mathbf{M}_4 + \mathbf{M}_2 \cdot \mathbf{M}_5 + \mathbf{M}_3 \cdot \mathbf{M}_6 + \mathbf{M}_4 \cdot \mathbf{M}_7 + \mathbf{M}_5 \cdot \mathbf{M}_8 + \mathbf{M}_6 \cdot \mathbf{M}_9\big) \\ &- J_F\big(3\,\mathbf{M}_1 \cdot \mathbf{M}_5 + 3\,\mathbf{M}_1 \cdot \mathbf{M}_6 + 3\,\mathbf{M}_2 \cdot \mathbf{M}_4 + 3\,\mathbf{M}_2 \cdot \mathbf{M}_6 \\ &\quad + 3\,\mathbf{M}_3 \cdot \mathbf{M}_4 + 3\,\mathbf{M}_3 \cdot \mathbf{M}_5 + 3\,\mathbf{M}_5 \cdot \mathbf{M}_7 + 3\,\mathbf{M}_6 \cdot \mathbf{M}_7 \\ &\quad + 3\,\mathbf{M}_4 \cdot \mathbf{M}_8 + 3\,\mathbf{M}_4 \cdot \mathbf{M}_9 + 3\,\mathbf{M}_6 \cdot \mathbf{M}_8 + 3\,\mathbf{M}_5 \cdot \mathbf{M}_9\big) \end{aligned}$$

In summary, the energy parameters, E_0 and all J's, can be obtained by solving the following matrix,

$$\begin{bmatrix} E_1^{FM} \\ E_1^{AFM} \\ E_2^{AFM} \\ \cdots \\ E_n^{AFM} \end{bmatrix} = \begin{bmatrix} 1 & w_1 & x_1 & \cdots & z_1 \\ 1 & w_2 & x_2 & \cdots & z_2 \\ 1 & w_3 & x_3 & \cdots & z_3 \\ \cdots & \cdots & \cdots & \cdots & \cdots \\ 1 & w_n & x_n & \cdots & z_n \end{bmatrix} \begin{bmatrix} E_0 \\ J_A \\ J_B \\ J_C \\ \cdots \\ J_n \end{bmatrix}$$

The corresponding magnetic configurations are presented in Figures S2.

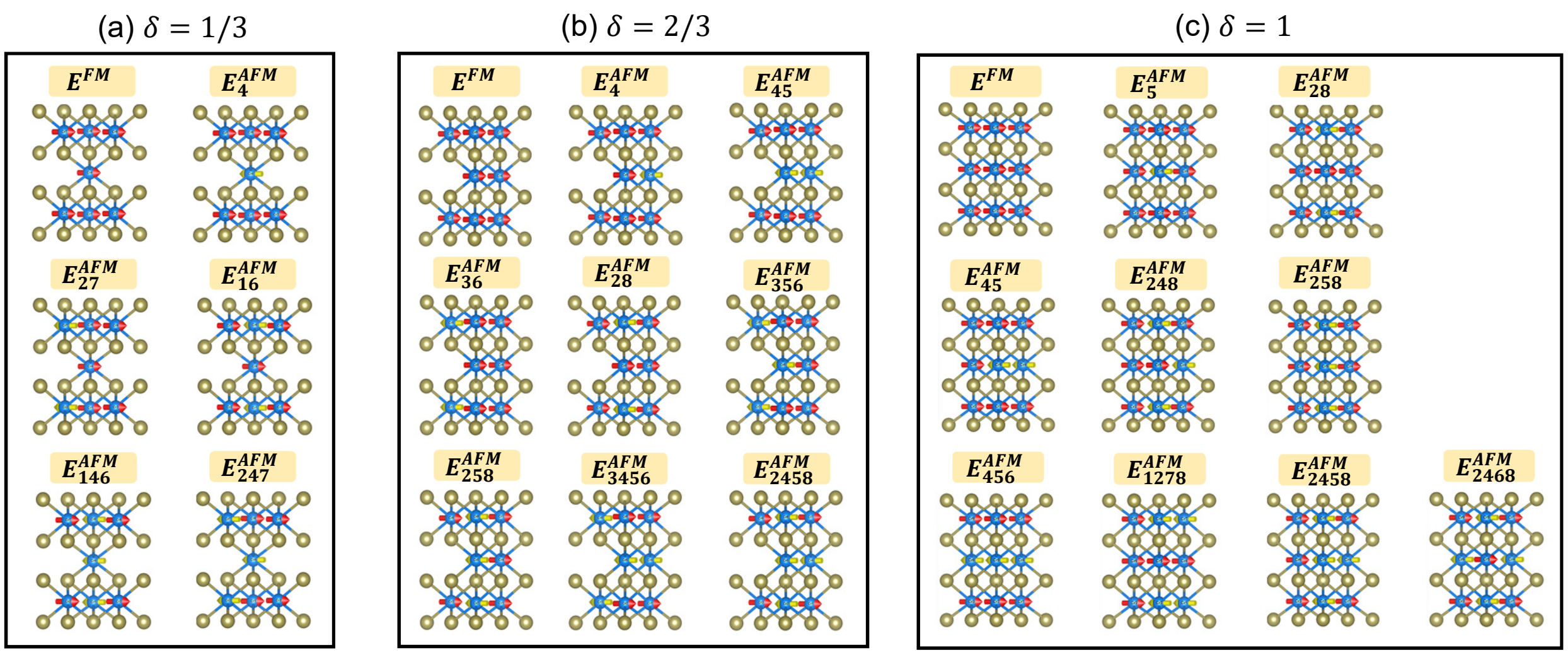

FIG. S2. Magnetic configurations to obtain the energy parameters of Heisenberg exchange model for $CrTe_2$-Cr_δ-$CrTe_2$ ultrathin films with δ = 1/3, 2/3, and 1.

S-IV. SPIN-TRANSFER ANALYSIS

To fully understand the interplay between the layer-resolved magnetic anisotropies, we introduce a layer-resolved spin-transfer analysis for all four $CrTe_2-Cr_\delta-CrTe_2$ ultrathin films ($\delta = 0$, 1/3, 2/3, and 1), schematically depicted in Figures S3(a)-(d). The DFT-SOT calculated spin accumulation torque $-\mathbf{\Phi}_i^{\mathrm{M}}$ on a given layer i is the sum of an incoming and an outgoing spin current flow (denoted by grey arrows). In the absence of spin current flow from the vacuum, we can simply set $a = h = 0$ for all cases, and subsequently determine the magnitude of each spin current flow (denoted by $b \sim g$). Through this analysis, we successfully identified the microscopic mechanism responsible for this complex behavior: a competition between two distinct interfacial SOT (iSOT) sources.

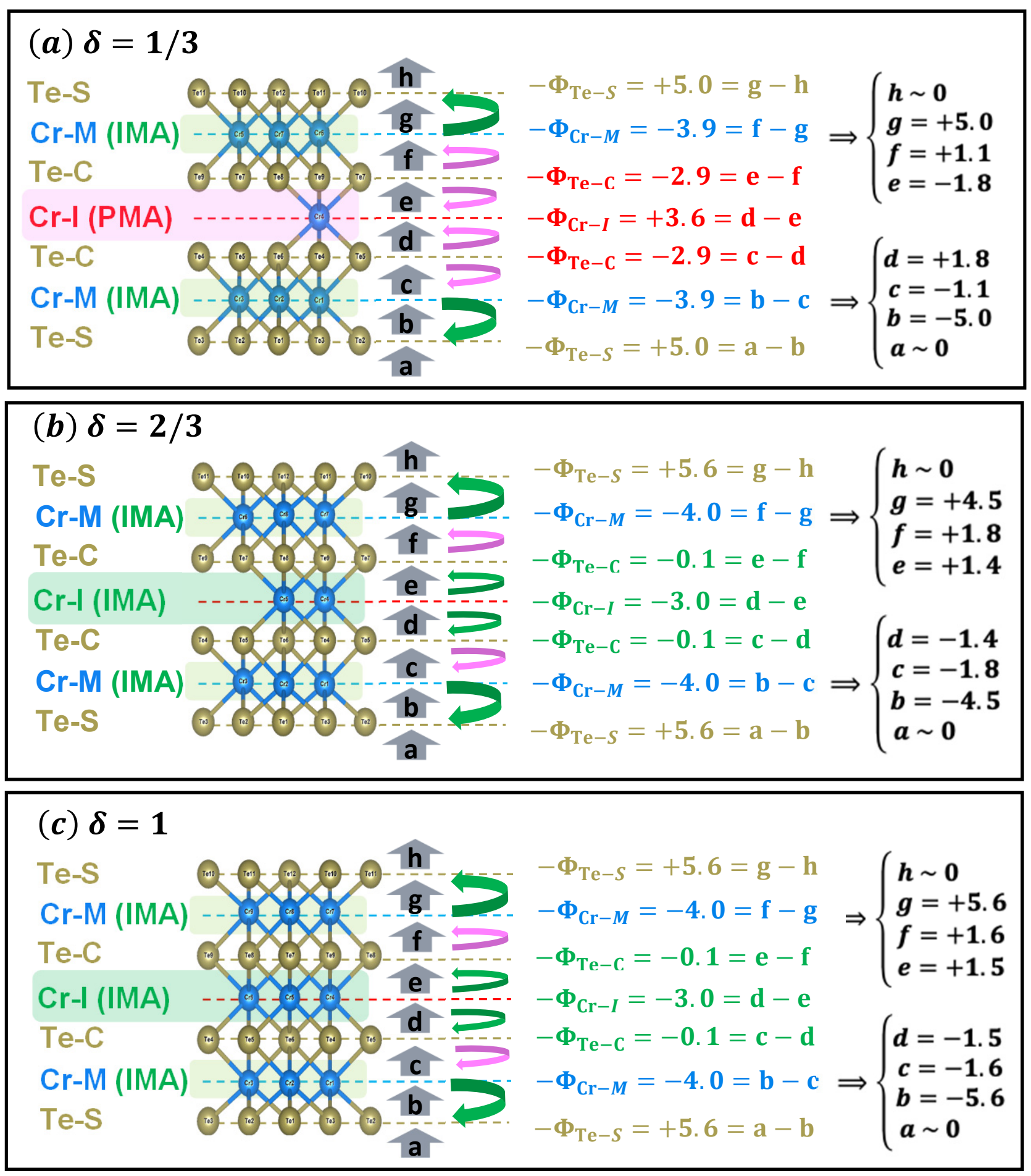

FIG. S3. Spin-Transfer Analysis for $CrTe_2$-Cr_δ-$CrTe_2$ ultrathin films with $\delta = 0$, 1/3, 2/3, and 1. The grey arrows, denoted by $a \sim h$, represent incoming and outgoing spin current flow at each atomic layer. The green (pink) curved arrows represent the spin transfer from the Cr (Te) to the Te (Cr) layers.

S-V. MICROMAGNETIC SIMULATION METHODOLOGY

Micromagnetic simulations were performed using the MuMax3 package [10] to investigate the magnetic properties of $CrTe_2-Cr_{1/3}-CrTe_2$ ultrathin films. The simulations involved sweeping an external magnetic field (H_{ext}) from +8 T to −8 T with a step size of 0.01 T. At each field step, the system was allowed to relax to its ground state using the *relax()* function to minimize the total system energy and determine the equilibrium magnetic configuration. Guided by our layer-resolved DFT-SOT calculations, three-, five-, and seven-layer models with two-dimensional periodicity in the xy-plane were constructed for the M2, M3, and M4 Cr_2Te_3 ultrathin films, respectively. Taking the M2 system ($\delta = 1/3$ in this study) as an example, the central Cr-I layer was modeled with perpendicular magnetic anisotropy (PMA), whereas the top and bottom Cr-M layers were initialized with in-plane magnetic anisotropy (IMA).

The computational domain was discretized into a $1 \times 1 \times 3$ grid with a cell size of $3.67 \times 3.67 \times 3.06$ Å^3. Notably, the fundamental micromagnetic parameters were mapped directly from our first-principles calculations. The saturation magnetization (M_{sat}), defined as the magnetic moment per unit volume, is calculated via:

$$M_{sat} = \frac{m}{V} \cdot 9.274 \times 10^{-24} ,$$

where m (μ_B) is the atomic magnetic moment, V (m^3) is the unit cell volume, and $1\ \mu_B = 9.274 \times 10^{-24}$ A $\cdot$ m^2.

The effective uniaxial magnetic anisotropy constant (K_u) for each Cr layer was strictly derived from the DFT-SOT magnetic torque evaluated at $\theta = 45°$ ($\mathbf{T}(\theta = 45°)$). The torque acting on the magnetic moments originates from the effective uniaxial anisotropy field (B_u), expressed as:

$$|B_u| = \frac{|\mathbf{T}(\theta = 45°)|}{m} \cdot \frac{1}{5.788 \times 10^{-5}} ,$$

where B_u is given in units of T, $\mathbf{T}(\theta = 45°)$ (eV) is the torque energy, m (μ_B) is the magnetic moment, and $1\ \mu_B = 5.788 \times 10^{-5}$ eV/T. Consequently, the magnetic anisotropy constant K_u, representing the energy density, is determined by:

$$K_u = \frac{1}{2} B_u \cdot M_{sat} = \frac{1}{2} \frac{|\mathbf{T}(\theta = 45°)|}{V} \cdot 1.6 \times 10^{-19} ,$$

where K_u is expressed by J/m^3, V (m^3) is the unit cell volume, and 1.6×10^{-19} is the conversion factor from eV to Joules. All corresponding parameters for the M2, M3, and M4 films are

comprehensively summarized in Tables S2–S4. The layer-resolved SOT components at $\theta = 45°$ for the M3 and M4 $CrTe_2-Cr_{1/3}-CrTe_2$ ultrathin films are presented in Figure S4.

To capture the interatomic exchange interactions in the continuum model, the micromagnetic exchange stiffness (A_{ex}) of the M2 $CrTe_2-Cr_{1/3}-CrTe_2$ films was mapped from the first-principles exchange coupling parameters (J) using the following relation:

$$A_{ex} = \frac{J \cdot m_1 m_2 \cdot d^2}{2V} \times 1.6 \times 10^{-19} ,$$

where A_{ex} (J/m) is the exchange stiffness, J (eV) is the exchange coupling energy, d (m) is the interatomic distance, and V (m^3) is the local volume of the Cr unit cell. To ensure dimensional consistency during the energy conversion (1 eV = 1.6×10^{-19} J), m_1 and m_2 are treated as the dimensionless magnitudes of the coupled magnetic moments (normalized by μ_B). Given that the spatial and magnetic moment fluctuations among the Cr atoms are negligible, the relation essentially scales as $A_{ex} \propto J \cdot d^2$.

To further simulate the competing exchange fields between the IMA Cr-M and PMA Cr-I layers, the individual exchange couplings were mathematically homogenized into effective intralayer (J_{intra}) and interlayer (J_{inter}) parameters. For J_{intra}, the effective parameter was evaluated by calculating the weighted average of J_A and J_B, governed by their bonding multiplicities and the squares of the interatomic distances:

$$J_{intra} = \frac{2J_A d_A^2 + J_B d_B^2}{2d_A^2 + d_B^2}.$$

Similarly, J_{inter} across the interface was formulated as:

$$J_{inter} = \frac{2J_E d_E^2 + J_D d_D^2}{2d_E^2 + d_D^2},$$

where the specific coupling parameters and interatomic distances are documented in Table S5. Finally, J_{intra} and J_{inter} were translated into A_{ex} to define the micromagnetic input parameters (see Table S5).

For the intralayer exchange stiffness, the positive J_A reinforces the likewise positive J_B, yielding a robust macroscopic average of $A_{intra} = 1.35 \times 10^{-12}$ J/m. In stark contrast, the strongly negative J_D competes with the positive (ferromagnetic) J_E, as clearly shown in Table S5. This atomic-scale frustration intrinsically suppresses the net macroscopic average, resulting in a significantly reduced interlayer stiffness of $A_{inter} = 1.30\times10^{-13}$ J/m. The physical consequence of this geometric frustration is a distinct energy hierarchy where the unfrustrated intralayer exchange is approximately

an order of magnitude stronger than the frustrated interlayer exchange. For analytical simplicity, the demagnetization field was neglected, and periodic boundary conditions were imposed in the film plane with a repetition vector of $(10, 10, 0)$.

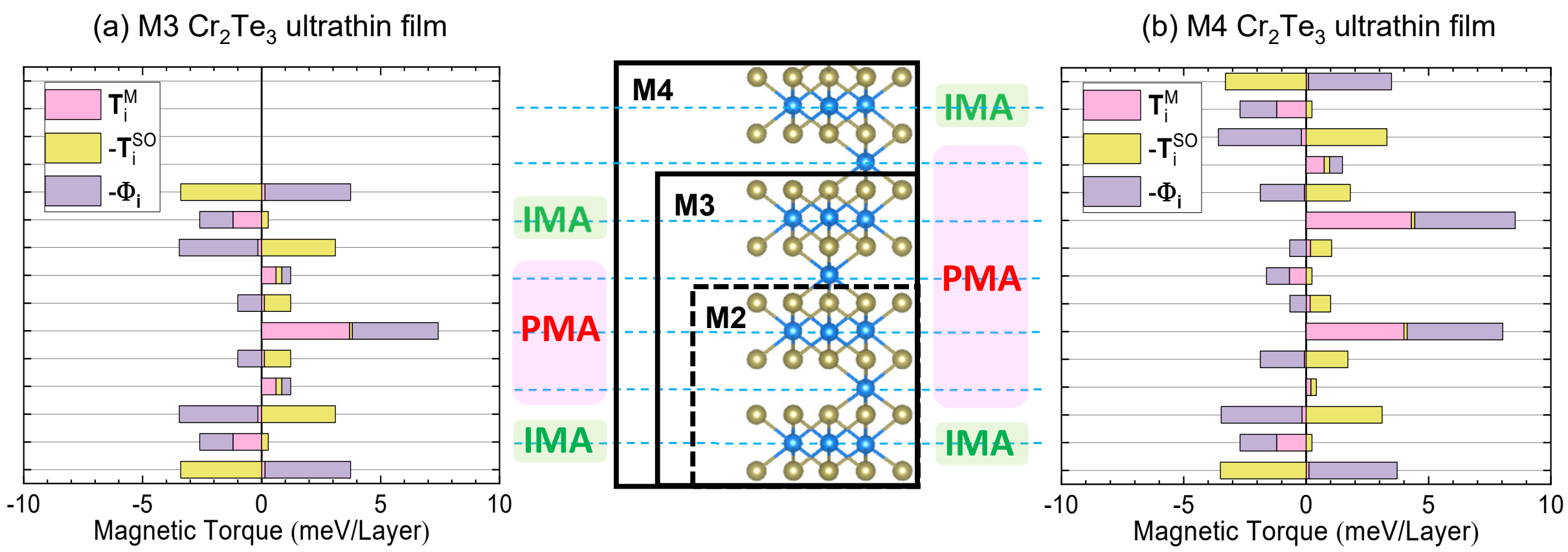

FIG. S4. Layer-resolved SOT components evaluated at $\theta = 45°$ for (a) M3 and (b) M4 $CrTe_2-Cr_{1/3}-CrTe_2$ ultrathin films. The net magnetic torque acting on layer i is decomposed as $\mathbf{T}_i^{\mathrm{M}} = -\mathbf{T}_i^{\mathrm{SO}} - \mathbf{\Phi}_i$. The pink and green shaded regions denote torque contributions favoring perpendicular (PMA) and in-plane (IMA) magnetic anisotropy, respectively.

Layers	M_{sat} (A/m)	K_u (J/m^3)	B_c (T)
Cr-M	7.07×10^5	-2.39×10^6	6.76
Cr-I	2.26×10^5	2.32×10^6	20.53
Cr-M	7.07×10^5	-2.39×10^6	6.76

TABLE S2. Layer-resolved micromagnetic parameters for the M2 $CrTe_2-Cr_{1/3}-CrTe_2$ ultrathin films derived from DFT-SOT calculations. The table lists the saturation magnetization (M_{sat}), the effective uniaxial magnetic anisotropy constant ($K_u = \mathbf{T}^{\mathrm{M}}(\theta = 45°)$), and the theoretical coercive field (B_c) evaluated using the relation $B_c = 2|K_u|/M_{\mathrm{sat}}$.

Layers	M_{sat} (A/m)	K_u (J/m^3)	B_c (T)
Cr-5	7.15×10^5	-7.80×10^5	2.18
Cr-4	2.40×10^5	3.96×10^5	3.30
Cr-3	7.35×10^5	2.40×10^6	6.54
Cr-2	2.40×10^5	3.96×10^5	3.30
Cr-1	7.15×10^5	-7.80×10^5	2.18

TABLE S3. Layer-resolved micromagnetic parameters (M_{sat}, K_u, and B_c) derived from DFT-SOT calculations for the M3 Cr_2Te_3 ultrathin films.

Layers	M_{sat} (A/m)	K_u (J/m^3)	B_c (T)
Cr-7	7.17×10^5	-8.06×10^5	2.25
Cr-6	2.40×10^5	1.30×10^5	1.08
Cr-5	7.41×10^5	2.62×10^6	7.06
Cr-4	2.50×10^5	-4.49×10^5	3.06
Cr-3	7.41×10^5	2.78×10^6	7.50
Cr-2	2.40×10^5	4.81×10^5	4.00
Cr-1	7.17×10^5	-7.86×10^5	2.19

TABLE S4. Layer-resolved micromagnetic parameters (M_{sat}, K_u, and B_c) derived from DFT-SOT calculations for the M4 Cr_2Te_3 ultrathin films.

Coupling	Path	J (meV)	d (Å)	J_{eff} (meV)	d_{eff} (Å)	m_1, m_2	A_{ex} (pJ/m)
Intralayer	J_A	1.25	3.86	1.40	3.86	3.10, 3.10	1.35
	J_B	1.70	3.86				
Interlayer	J_D	−1.75	3.06	0.22	3.06	3.01, 3.10	0.13
	J_E	0.60	4.90				

TABLE S5. Multi-scale mapping of the exchange coupling parameters from first-principles calculations to micromagnetic simulations. The table details the homogenization of individual microscopic exchange paths (J_A, J_B for intralayer; J_D, J_E for interlayer) and their respective interatomic distances (d) into effective coupling parameters (J_{eff}). These homogenized values are subsequently converted into the continuum exchange stiffness (A_{ex}) using the effective projection distance (d_{eff}) and the dimensionless magnitudes of the coupled localized magnetic moments (m_1, m_2, normalized by μ_B). The local volume of the Cr unit cell is fixed at $V = 118.52$ Å^3 for all energy conversions.

[1] M. C. Payne, M. P. Teter, D. C. Allan, T. Arias, and a. J. Joannopoulos, Iterative minimization techniques for ab initio total-energy calculations: molecular dynamics and conjugate gradients, Reviews of modern physics **64**, 1045 (1992).

[2] G. Kresse and J. Furthmüller, Efficient iterative schemes for ab initio total-energy calculations using a plane-wave basis set, Physical review B **54**, 11169 (1996).

[3] G. Kresse and D. Joubert, From ultrasoft pseudopotentials to the projector augmented-wave method, Physical review b **59**, 1758 (1999).

[4] J. Taylor, H. Guo, and J. Wang, Ab initio modeling of quantum transport properties of molecular electronic devices, Phys. Rev. B **63** (2001).

[5] D. Waldron, L. Liu, and H. Guo, Ab initio simulation of magnetic tunnel junctions, Nanotechnology **18**, 424026 (2007).

[6] B.-H. Huang, Y.-F. Lai, and Y.-H. Tang, Validity of DFT-based spin-orbit torque calculation for perpendicular magnetic anisotropy in iron thin films, AIP Advances **13**, 015034 (2023).

[7] B.-H. Huang, Y.-H. Fu, C.-C. Kaun, and Y.-H. Tang, Determining perpendicular magnetic anisotropy in Fe/MgO/Fe magnetic tunnel junction: A DFT-based spin–orbit torque method, Journal of Magnetism and Magnetic Materials **585**, 171098 (2023).

[8] Y. Zhong, C. Peng, H. Huang, D. Guan, J. Hwang, K. H. Hsu, Y. Hu, C. Jia, B. Moritz, D. Lu, J.-S. Lee, J.-F. Jia, T. P. Devereaux, S.-K. Mo, and Z.-X. Shen, From stoner to local moment magnetism in atomically thin cr2te3, Nature Communications **14**, 5340 (2023).

[9] W. Pan, C. Xu, X. Li, Z. Xu, B. Liu, B.-L. Gu, and W. Duan, Chiral magnetism in lithium-decorated monolayer $crte_2$: Interplay between dzyaloshinskii-moriya interaction and higher-order interactions, Phys. Rev. B **109**, 214405 (2024).

[10] A. Vansteenkiste, J. Leliaert, M. Dvornik, M. Helsen, F. Garcia-Sanchez, and B. Van Waeyenberge, The design and verification of mumax3, AIP Advances **4**, 107133 (2014).
